# International Hahnemannian Association, Annual Meeting of 1891

**Published:** 1891-11

**Authors:** 


### INTERNATIONAL HAHNEMANNIAN ASSOCIATION, ANNUAL MEETING OF 1891.

(Special Reports.)

### First Day's Session.

Tuesday Morning, June 23d, 1891, 11 A. M.

### REPORT OF CORRESPONDING SECRETARY.

Dr. W. P. Wesselhœft—*Mr. President and Ladies and Gentlemen:* I, as the Corresponding Secretary, have not corresponded with anybody, but necessarily, on account of my health, have had to go to Europe this spring. During a short sojourn there, I took advantage of the opportunity to meet with what homœopathic doctors I could, talk with them concerning Homœopathy, tell them about our Society here, and what we were doing in this country, and in my interviews I came across some things of interest. I have been home only a few days, and have had no time to make out a written report, but will just give you a short verbal account of what I saw there.

I took every opportunity to let them know that such a society as the *International Hahnemannian Association* existed, a fact of which they were, with one exception, ignorant. Indeed, the widespread ignorance there of the standing of Homœopathy in America was astounding. I told them of our work here, and to those men whom I thought would make good use of it I gave a copy of our Transactions.

My first visit was in Leipsic, upon Dr. Lorbacher, who received me kindly and invited me to go with him to the hospital which he has there. It has been endowed recently—that is, within the last ten years—sufficiently to enable them to put a wing on an old villa and turn it into an exceedingly good hospital. They have accommodation for sixty beds there, but there were only about ten patients. The reason of this paucity of patients was, he told me, on account of a very peculiar institution in force among the poorer classes—laborers, mechanics, and so on—called *Krankenkasse.* Each member pays a small amount of money into a bank regularly, and for this stipend receives medical treatment. It is under the control of physicians who are appointed as district physicians of the town.

Up to the present time no homœopath has been appointed on that commission, but this year two homœopaths have been appointed in Leipsic. I found in the hospital the usual way of treating, and the lower potencies mostly in use. Lorbacher says he gives the thirtieth, but the others give the sixth to twelfth in rapidly repeated doses. In their pharmacy I saw some things which were not exactly homœopathic.

The saddest thing I saw there was a patient still lingering and suffering from the injection of the Koch lymph. I expressed my indignation as strongly as I could before the young men and the assistants, and I think they were all terrifically ashamed of it.

It happened that the day before, in the German Parliament, an appropriation was asked for the purpose of extending the benefits of the Koch lymph more generally, and then Professor

Virchow told the blunt truth; not a single cure has been effected, said he, but hundreds have through its agency suffered the torments of the damned.

I went to Weimar, and saw my old friend Goullon, who has almost retired from practice. I was there told of a Dr. Gœtze, upon whom I called, but at first found him out on a trip to a neighboring city. What I heard of him was of the very best, and I had no doubt of his being a true follower of Hahnemann. I left him, as I had left Dr. Lorbacher, two copies of our Transactions. Dr. Gœtze is a good English scholar, and was entirely ignorant of what we were doing in this country.

I told these men of the great improvements in our homœopathic literature. They both said that they would immediately subscribe for the *Medical Advance* and THE HOMŒOPATHIC PHYSICIAN.

In Munich I was in hopes of meeting Koch, but he had gone on a vacation, and, as my time was limited, I did not have the pleasure of meeting him.

In Italy, which was my objective point, I went as far as Verona. I indulged in the hope of meeting our member, Dr. Pompili, but I did not have that pleasure. As I had never been in Rome in my life, I thought I would leave it for some other time when I could see Rome and Pompili at the same time. In Italy I could not find anybody who knew anything about Homœopathy at all, and I could not have talked with them if I had.

In Frankfurt I called on Zimrock, an active practitioner with a large business. He approached me immediately with a case for which he wanted me to tell him the remedy. He gave me a lot of pathological symptoms, that it would be impossible for any one to prescribe on, and I told him so. He said, "O ho! you are one of those fellows, are you; you must go to see Dr. Sægert." So I did, and I found him a most interesting man. By the way, I did not leave Zimrock any of the Transactions.

I found, in Dr. Sægert, a most remarkable man. He had formerly been a surgeon in the German army, and was now practicing Homœopathy in the city of Frankfurt. I spent a most charming evening with him. I might say night, for we talked pretty late. He was perfectly astonished when I told him about what we were doing here and of the strength in numbers and in purpose of the true advocates of Homœopathy in this country. I left him the Transactions and he said he would become a subscriber for our journals, and also a member of the I. H. A. Above everything he wanted the highest potencies; it was perfectly wonderful to him. In Cologne my stay was very short, and I met nothing of interest to you. In Bremen I found one homœopath only, Dr. Antze. He was a scholarly man, the friend and successor of Dr. Krummacher, one of the pioneers of Homœopathy in Germany, who died only a few weeks before I arrived. Dr. Antze has a large and very influential practice; he is frequently called to Berlin in consultation, at which place there is no homœopath worthy of the name. He also received the Transactions and will become a member of our Society, will send for high potencies and subscribe for our journals. I found his wife was almost as good a homœopath as he was.

The only school abroad where anything like genuine Homœopathy can be learned is at Budapest, largely under the control of Dr. Badoky, of whom I heard good accounts, though I did not see him. A disadvantage under which homœopaths labor in some parts of Germany is that they are obliged by law to send to the shops for their prescriptions. Lowerbach told me he had to send to the pharmacies for his milk-sugar, but not for medicine. In Prussia they have the liberty of dispensing their own medicines, but not in Saxony or Bavaria. In those districts they still have to send to the apothecary shops. Every physician that I saw looked to America as the hope of Homœopathy, although I do not think that it has gone back or diminished in Germany. They have about held their own. This is about the report I have to make as Corresponding Secretary, although there is no correspondence about it.

### REPORTS OF DELEGATES.

Dr. Kennedy—I scarcely expected to be called on to make a report, in view of the fact that I am only an infant in the Association, indeed scarcely born yet. It gives me pleasure to report that we have a little society called the Boston Hahnemannian Association, and the name is significative of our object and purpose. It is the outgrowth of a still smaller club formed for mutual benefit and associated study. By enlarging our borders and taking in others we hope to do a share in the work of promulgating the principles of Hahnemann. We number altogether twenty-five, our meetings are monthly, and are presided over by Dr. R. L. Thurston, a staunch homœopath, and a strong man.

Our method of conducting the meeting is this: we devote a portion of the evening to the study of the *Organon*, reading certain sections and discussing them. The remainder of the time is taken up by clinical verifications. Cases are reported, not particularly interesting, perhaps, as cases, but as verifying some particular symptoms as being removed by a remedy. We endeavor to make the reports, regardless of the disease and not for the case *per se*, but simply as a verification of some portion of the materia medica. In this way we hope to improve our knowledge and our skill in making prescriptions. We also report new symptoms that have been removed by a remedy, but do not incorporate them until they have been thoroughly verified. It has been a great help to all of us, and we hope Homœopathy will be aided by these studies.

Dr. J. H. Allen—We have done quite a good work in Indiana this year. Dr. Sawyer, in his address as President, greatly encouraged us; among the many valuable suggestions offered by him was one that we should form societies for the study of the *Organon*, which we hope to see effected. He also presented able arguments why our school should be represented in the public institutions of our State, and a committee was appointed to see the Legislature. Most of our Bureaus have members on them who are pure homœopaths. For the first time we have had a stenographer this year who took a report of the discussions. The papers and the discussions will be published, and will form quite a large volume.

Dr. Carr—The Rochester Hahnemann Society was organized about five years ago by a few members of the Monroe County Society. They met for the avowed purpose of reading and studying the *Organon*. From three or four members we have grown to nine. It has had a great deal to contend with, as some of the members of the Monroe County Society have taken great pains to hamper us. Still, we see signs that the laity are beginning to appreciate that there is a difference between the so-called Homœopathy and the Homœopathy of Hahnemann.

Dr. Powel—I represent the Organon and Materia Medica Society, and, as our name indicates, we are students of the *Organon*, materia medica, and clinical medicine. We have an active membership of twenty-five and an honorary membership of twelve. From our members originated the Post-Graduate School of Homœopathics, of which you will hear more during this session.

Dr. Rushmore—I have no particular report to spread before you. I will say, however, that genuine Homœopathy is growing stronger in our Society every year.

Dr. J. B. G. Custis—We have in Washington a hospital under homœopathic management, and, while I cannot claim that nothing but Hahnemannian Homœopathy is practiced there, I do claim that it is practiced there more and more each year, and that the gentlemen there are more careful in their prescriptions, and there is far less odor of Iodoform and other disinfectants than formerly, and our reports of cures increase correspondingly. We take all kinds of cases regardless of the prognosis.

I have been criticised because I allowed myself to be placed on the staff of a hospital where anything but true Homœopathy is practiced, and in this regard I want to make the point that the best way to improve the practice and therapeutics of hospitals is for the physicians belonging to this Association to get on the staff of as many hospitals as they can. As we cannot often get hospitals over which we have exclusive control, this is the best policy to pursue. We can thereby best show the difference in the effects of the two treatments. We thus do good missionary work, which is one of the objects of this Association.

Dr. Fincke—I represent the Homœopathic Union of New

York. It is an *Organon* society; we have now existed for a year, going through the *Organon*, and have arrived only to 110 section, which shows that we do it pretty thoroughly. We invite, by postal cards, any one who desires to study the subject to come to our meetings, and we have done some very profitable work. Those who have attended regularly have derived a great deal of benefit from it. Such societies should be in every city, not necessarily organized in a formal manner, but just simply in a social way to read one or two sections and talk it over.

Dr. Cowley—The Farrington Club, of Allegheny County, had existed for about four years, when, on returning from the I. H. A. meeting last year, I proposed that it should be disbanded and an *Organon* club formed in its stead. It was not carried, but we agreed to read the *Organon* as the Farrington Club, and it is possible that as we improve we may come out as a full-fledged *Organon* society in time.

